# BnaA.bZIP1 Negatively Regulates a Novel Small Peptide Gene, *BnaC.SP6*, Involved in Pollen Activity

**DOI:** 10.3389/fpls.2017.02117

**Published:** 2017-12-12

**Authors:** Xuanpeng Wang, Xin Li, Mengmeng Li, Jing Wen, Bin Yi, Jinxiong Shen, Chaozhi Ma, Tingdong Fu, Jinxing Tu

**Affiliations:** National Key Laboratory of Crop Genetic Improvement, National Subcenter of Rapeseed Improvement in Wuhan, Huazhong Agricultural University, Wuhan, China

**Keywords:** *Brassica napus*, small peptide, *BnaC.SP6*, pollen-specific, *BnaA.bZIP1*, transcriptional repressor

## Abstract

Small peptides secreted to the extracellular matrix control many aspects of the plant’s physiological activities which were identified in *Arabidopsis thaliana*, called ATSPs. Here, we isolated and characterized the small peptide gene *Bna.SP6* from *Brassica napus*. The *BnaC.SP6* promoter was cloned and identified. Promoter deletion analysis suggested that the -447 to -375 and -210 to -135 regions are crucial for the silique septum and pollen expression of *BnaC.SP6*, respectively. Furthermore, the minimal promoter region of p158 (-210 to -52) was sufficient for driving gene expression specifically in pollen and highly conserved in *Brassica* species. In addition, *BnaA.bZIP1* was predominantly expressed in anthers where *BnaC.SP6* was also expressed, and was localized to the nuclei. BnaA.bZIP1 possessed transcriptional activation activity in yeast and protoplast system. It could specifically bind to the C-box in p158 *in vitro*, and negatively regulate p158 activity *in vivo*. BnaA.bZIP1 functions as a transcriptional repressor of *BnaC.SP6* in pollen activity. These results provide novel insight into the transcriptional regulation of *BnaC.SP6* in pollen activity and the pollen/anther-specific promoter regions of *BnaC.SP6* may have their potential agricultural application for new male sterility line generation.

## Introduction

In flowering plants, pollen grains, which are required for successful fertilization, are formed by meiosis in microsporocyte and mitosis in pollen (McCormick, 2004; Twell, 2011). The seeds and fruits derived from double fertilization of flowering plants are major components of human diet. With the increasing human population and changes in the global climate, breeders are faced with the challenge of developing new hybrid varieties for sustained food supply (Pachauri et al., 2014). In F1 hybrid seed production, the “two-line system” has shown a greater potential after the “three-line system” reached a yield plateau (Cheng et al., 2007). This observation has also been made in *Brassica napus*, the hybrid seeds of which are widely used commercially in China. Male sterility through genetic engineering is the most effective strategy for improving yields by producing fertile F1 hybrids (Ananthi et al., 2013). This can be achieved by inhibiting the normal endogenous hormone biosynthesis or by combining the pollen/anther-specific promoter with the genes of appropriate enzymes or toxin proteins so as to restrict the development of reproductive tissues (Bae et al., 2010; Xia et al., 2016). Thus, identification of the pollen/anther-specific promoter is necessary for successful genetic manipulation. Many pollen/anther-specific promoters have been cloned and characterized from various plant species; these include the *SBgLR* promoter from potato, *OSIPA* promoter from rice, and *Zm908* promoter from maize (Lang et al., 2008; Swapna et al., 2011; Peng et al., 2017). Moreover, some *cis*-acting regulatory elements have been delineated by deletion scanning and by changing the *cis*-acting elements (Shiba et al., 2001; El-Shehawi et al., 2010), such as the POLLEN1LELAT52, GTGA MOTIF, and TTTCT (Eyal et al., 1995; Rogers et al., 2001; Chang et al., 2017). However, the presence of these pollen-specific *cis*-elements does not equal to a promoter’s pollen specificity (Hamilton et al., 1998). Only a few *trans*-acting factors, such as atDUO1, γMYB1 and γMYB2, and ZmDof30, which interact with the pollen-specific promoters, have been confirmed (Borg et al., 2011; Nguyen et al., 2016; Peng et al., 2017).

Plant bZIP transcription factors (TFs) are characterized by a leucine zipper and a basic region, which is responsible for the specific binding to various ACGT-containing elements in the promoters (Izawa et al., 1993). They are classified as A-box, C-box, G-box, or T-box according to the nucleotide at position +2 (the central two nucleotides C and G are designated as -0 and +0, respectively), among which C and/or G-boxes are preferentially bound by plant bZIP proteins (Schumacher et al., 2000). In *Arabidopsis*, 75 bZIP TFs were subdivided into 10 groups named A to I, and S, based on sequence similarities and functional features (Jakoby et al., 2002). Gibalova et al. (2017) identified 17 bZIP genes possessing a mean expression signal in pollen over 400 in the Affymetrix Arabidopsis ATH1GeneChip, including *AtbZIP1*, *AtbZIP18*, *AtbZIP34*, *AtbZIP52*, and *AtbZIP61*. Furthermore, they demonstrated that AtbZIP18 interacted with AtbZIP34, AtbZIP52, and AtbZIP61 in Y2H assays (Gibalova et al., 2017). The pollen of atbzip18 showed similar morphological defects but with different percentage compared to atbzip34 pollen which appearing misshapen and misplaced nuclei in the cytoplasm (Gibalova et al., 2009). Further pollen microarray analysis indicated that they are functional redundancy in pollen and the potential pollen-expressed repressor role of AtbZIP18. *AtbZIP1*, an S-group member of bZIP TFs, was confirmed to be expressed highly in various tissues including pollen and silique valve. AtbZIP1 is involved in sugar signaling, nutrient signaling, protein network integration, and DNA binding (Kang et al., 2010; Para et al., 2014). These facts imply that their *Brassica* ortholog genes, *BnbZIPs*, may be involved in the transcriptional regulation network of pollen development in *B. napus*.

Many genes encoding putative small peptides have been identified in plants by genomic study and multi-omics analysis in recent years (Fukuda and Higashiyama, 2011; Nakamura et al., 2012; Huang et al., 2015). In maize, *Zm908p11*, a gene predominant in pollen, was identified; this gene encodes a 97-amino-acid (a.a.) peptide that functions in pollen tube growth as a profilin ligand (Dong et al., 2013). In *Arabidopsis*, 152 putative small secreted protein genes that encode proteins possessing a signal peptide at N terminal, and are composed of less than 100 a.a. residues, are defined as ATSPs. One unannotated ATSP member, *ATSP6*, was demonstrated to express weakly in root elongation zone and meristem by promoter-GFP analysis (Nakamura et al., 2012). Based on e-FP Browser data, *ATSP6* exhibits high expression levels in mature pollen and encodes a protein that is yet unidentified.

As of date, no studies have reported the biological function of *Bna.SP6* in *B. napus*, which is a homolog of *ATSP6*. In this study, we characterized the important regions in pBnaC.SP6 and identified a *trans*-factor of *BnaC.SP6* using different assays. Our findings could help in better understanding of the regulation of *BnaC.SP6* at the transcription level in pollen activity.

## Materials and Methods

### Plant Materials

*Brassica napus* “ZS11” and a near-isogenic line “S45AB” were sown at the experimental station of Huazhong Agricultural University (Wuhan, China) under natural conditions. *Arabidopsis thaliana* Col-0 was grown in plastic pots containing soil mixture (nutrient soil:roseite = 3:1) in a greenhouse at 22°C and under 16-h/8-h light/dark photoperiod.

### RNA Isolation and RT-PCR

Leaves, stems, whole inflorescence, flower buds (1.5, 2.5, 3.5, 4.5, 5.5, and 6.5 mm), opening anthers, and siliques (2, 11, and 29 dap) were collected from ZS11 plants; only buds without stamens were collected from S45A plants. Total RNA was extracted using RNeasy Plant Mini Kit (Qiagen, United States). The first-strand cDNA was then synthesized by reverse transcription and *Bna.SP6* transcript was amplified using Bna.SP6-F and Bna.SP6-R primers (Supplementary Table S1) with 32 cycles. Ubiquitin-associated (UBA) protein gene *BnUBA* (Yang et al., 2014) was amplified as a control (Supplementary Table S1).

### Gene Cloning and Sequence Analysis

The genomic DNA of BnSP6_C08 (611 bp) and BnSP6_A08 (475 bp), the pBnaC.SP6 (1167 bp), and the full-length CDS of *Bna.SP6* (246 bp) was amplified with specific primers (Supplementary Table S1) and sequenced. The *Bna.SP6* CDS sequences were translated to their respective peptide sequences using Primer Premier 5 software. The potential *cis*-acting elements in pBnaC.SP6 were predicted using the PlantCARE program (Lescot et al., 2002). The presence and location of signal peptide cleavage sites in the amino acid sequences were predicted by SignalP 4.1 server (Petersen et al., 2011).

### Construction of Promoter Reporter Plasmids

All the plasmids used for GUS assay were constructed in the backbone of pCAM2300-H2BYFP-gusplus-Nost vector. The H2BYFP-GUSplus-Nost fragment was restricted from pG2NHL-H2BYFP-GUSplus-Nost plasmid by *Kpn*I and *EcoR*I and ligated into pCAM2300.

A series of fragments with 5′-deletion in the promoter were amplified using different forward primers (p647F, p447F, p375F, p306F, p210F, and p135F) and a single reverse primer, Pro-1R. The full-length promoter and six 5′-deleted derivatives were cloned at *Sal*I and *Sma*I sites in the pCAM2300-H2BYFP-GUSplus-Nost vector and the resulting constructs were designated as pBnaC.SP6, p647, p447, p375, p306, p210, and p135, respectively. In addition, two 3′-deletions containing the regions from -306 to -52 and -210 to -52 were amplified using two forward primers (p306F and p210F) from the upstream region and a single reverse primer (Pro-d52R) from the downstream region. They were cloned into the pCAM2300-H2BYFP-GUSplus-Nost vector and the resulting constructs were designated as p254 and p158. The primers used are shown in Supplementary Table S1.

### Stable Transformation of *Arabidopsis* and Segregation Analysis of Transgenic Plants

Col-0 plants were transformed with recombinant *Agrobacterium tumefaciens* GV3101 strain, harboring the promoter:H2BYFP-GUSplus construct, by floral dip method (Clough and Bent, 1998). Seeds from the wild-type were germinated on agar plates containing half-strength Murashige and Skoog’s medium (12 MS), with 1% (w/v) sucrose, 0.7% (w/v) agar at pH 5.8, and supplemented with kanamycin (50 mg l^-1^) and timentin (75 mg l^-1^). Thereafter, we randomly selected 100–160 seeds of each T_1_ line to perform the segregation analysis. The kanamycin-resistant lines showing 3:1 segregation pattern were further carried onto the next generation to obtain homozygous lines. Three such homozygous lines were used for quantitative GUS activity assay. Chi-square values were calculated by the corrected formula $\chi_{C}^{2}={\Sigma \left(O-E-1/2\right)}^{2}/E$, where *O* and *E* are the observed and expected values, respectively. The probability was calculated with two-degrees-of-freedom based on Chi-square distribution table.

### Histochemical GUS and DAPI Staining Assays

The histochemical GUS and 4′,6-diamidino-2-phenylindole (DAPI) staining assays were performed as described by Luo et al. (2012) and Liu et al. (2017), respectively. Different tissues from heterozygous transgenic lines were stained overnight in X-Gluc solution. Thereafter, the chlorophyll was cleared out of the samples using 70% ethanol. Images of various tissues were taken using an Olympus DP72 Digital Microscope Camera.

GUS-stained flower buds were embedded in Technovit 7100 resin, as described previously by Zhu et al. (2010). Subsequently, transverse sections of the anthers (approximately 8 μm thick) were cut from the embedded blocks using a Leica Ultracut R ultramicrotome. The GUS-stained anthers were incubated at 60°C for 1 h in a DAPI staining solution containing 20% methanol and 1.0 μg ml^-1^ of DAPI. The images were taken using a Nikon ECLIPSE 80i microscope.

### Quantification of GUS Activity

*Arabidopsis* flower buds (flower development stage 12–15) of homozygous transgenic lines were collected for quantitative measurement of GUS activity according to the protocol described by Jefferson et al. (1987). Total proteins were extracted from the buds and the protein concentration was determined by the Bradford method (Bradford, 1976). The GUS assay buffer containing the substrate, 4-methylumbelliferyl-β-d-glucuronide (MUG), was added to the protein samples and the reaction was incubated for 30 min at 37°C. The resulting fluorescence was recorded using Tecan Infinite M200 PRO (Tecan Group Ltd., Switzerland). The relative GUS activities were calculated and expressed as nmole 4-MU generated per min per milligram of the total protein.

### Yeast One-Hybrid and Transcriptional Activation Assay in Yeast

A Y1H assay was performed as mentioned in the manual of Matchmaker Gold Yeast One-Hybrid Library Screening System (Clontech). As the baits, pAbAi-p158, pAbAi-mp158, and pAbAi-p99 (part of p158, containing only the ABRE motif) were transformed into the Y1HGold yeast strain. Then the pGADT7-BnaA.bZIP1 fusion plasmid was introduced into three bait strains, respectively. The co-transformed yeast cells were cultured on SD/-Leu agar plates with or without AbA and incubated at 30°C for 3 days. p53 was used as a positive control.

pGBKT7-BnaA.bZIP1 and pGBKT7-atbZIP1 fusion plasmids were introduced into yeast reporter strain AH109 (Clontech). The transformants were transferred onto a filter paper and incubated at 30°C for 3–5 h in the presence of X-Gal to check the β-galactosidase activity by monitoring the generation of blue color. The primers used are shown in Supplementary Table S1.

### Electrophoretic Mobility Shift Assay

The pET-32a-BnaA.bZIP1 recombinant plasmid was transformed into *Escherichia coli* BL21 cells. The recombinant fusion proteins were purified using Ni-NTA His•Bind^®^ Resin (Novagen). The complementary oligonucleotides (p59 and mp59) containing the consensus DNA-binding site C-box (CACGTC) and mC-box (Ctatga) were, respectively, annealed and used as DNA probes. The DNA–protein binding reactions were performed in a total volume of 10 μl, containing 5 μl BnaA.bZIP1-His or pET-32a-His protein, 2 μl 5× EMSA/Gel-shift-Binding Buffer (Beyotime Biotechnology, China), 30 nM Cy5-labeled probe, and 200- to 800-fold molar excess of unlabeled competitor. The reaction mixture was incubated for 30 min at 25°C. The electrophoresis was performed with 6% non-denaturing polyacrylamide gel and carried out in 0.5× TBE (45 mM Tris base, 45 mM boric acid, 0.5 mM EDTA, pH 8.3) at 4°C in a vertical electrophoresis system. The reaction mixture was loaded and electrophoresis was performed at 10 mA until the dye front migrated through 50% of the length of the gel. The gels were scanned to detect the fluorescent DNA using Fujifilm FLA-9000 plus DAGE (FujiFilm, Japan). The primers used are shown in Supplementary Table S1.

### Analyses of BnaA.bZIP1 Transcriptional Activation/Repression and DNA Binding in *Arabidopsis* Protoplasts

The dual luciferase reporter (DLR) assay was performed as described by Hao et al. (2010). The full-length BnaA.bZIP1 (139 a.a.), two C-terminal deletions (BnaA.bZIP1ΔC1, 114 a.a. and BnaA.bZIP1ΔC2, 89 a.a.) of BnaA.bZIP1, and AtbZIP1 (145 a.a.) restricted with *Xba*I and *Bam*HI were inserted into pBDGAL4 vector as effectors. Luciferase (LUC) driven by the CaMV35S promoter was used as a reporter. The *Renilla LUC* gene driven by the *Arabidopsis* UBIQUITIN3 (AtUBI3) promoter was used as an internal control.

In addition, the pGreenII 62-SK and pGreen II 0800-LUC transient expression system was employed as described by Hellens et al. (2005). *BnaA.bZIP1* restricted with *Bam*HI and *Xho*I was inserted into the pGreenII 62-SK vector as the effector. The fragments p158, mp158, p103 (part of p158, containing only the C-box), and mp103 restricted with *Sal*I and *Sma*I were inserted into the pGreen II 0800-LUC vector as reporters. The effector and reporter constructs were co-transformed into *Arabidopsis* protoplasts by polyethylene glycol (PEG)/calcium-mediated transformation (Yoo et al., 2007). After transfection, the luminescence from firefly *LUC* and *Renilla LUC* were recorded on a Tecan Infinite M200 PRO. The primers used are shown in Supplementary Table S1.

### Expression Pattern and Subcellular Localization of BnaA.bZIP1

The *BnaA.bZIP1* transcript was detected by quantitative real-time PCR (qRT-PCR) on a CFX96 Real-Time System (Bio-Rad, United States). The expression data were calculated using the 2^-ΔΔ*C*_t_^ method. Each sample was assayed in triplicate. The 1317-bp promoter region (pBnaA.bZIP1) of *BnaA.bZIP1* restricted with *Sal*I and *Bam*HI was inserted into the pCAM2300-H2BYFP-GUSplus-Nost vector.

The pM999-CFP-GHD7 plasmid was used as a nuclear marker. The full-length CDS of *BnaA.bZIP1*, without the termination codon, restricted with *Xba*I, was inserted into the pM999-GFP vector. The preparation of *Arabidopsis* mesophyll protoplasts and their subsequent transfection was done as described above. The fluorescence signals were measured using a FV1200 Laser Scanning Microscope. The primers used are shown in Supplementary Table S1.

### Dexamethasone (DEX) Treatment

The fusion protein of full-length BnaA.bZIP1 with a 3XFlag-tag at the N-terminal was cloned into pTA7002 vector (Aoyama and Chua, 1997). The vector construct was transformed into p158 transgenic line. T_2_ plants harboring BnaA.bZIP1 and p158 fragments were grown on soil and treated with DEX (Sigma–Aldrich, United States) at the flowering stage. Water (10 ml) containing 15 nM DEX was applied daily for 5 days to the soil. The day of first DEX treatment was recorded as 1 days. Buds (with p158 activity) were collected from T_2_ plants on 0, 4, and 6 days after the DEX treatment. RNA extraction and qRT-PCR were performed as described above and Actin7 was used as the reference. The primers used are shown in Supplementary Table S1.

## Results

### Small Peptide Gene *BnaC.SP6* Was Expressed in Mature Anthers and Silique Septum

**FIGURE 1:**
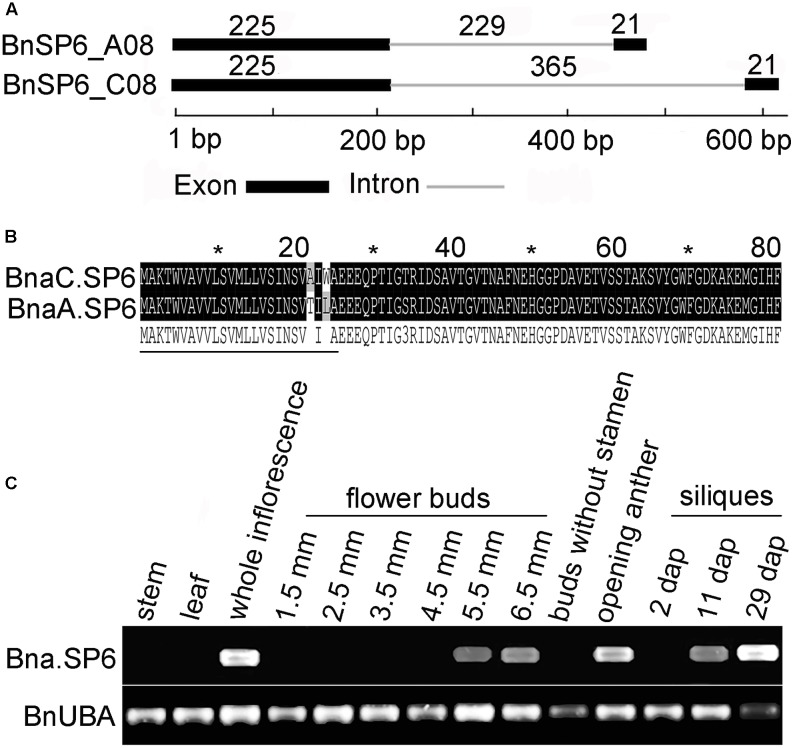
Structural analysis of the small peptide gene *Bna.SP6* in *Brassica napus*. **(A)** Schematic of BnSP6_C08 and BnSP6_A08 gene structures. Exons and intron are indicated by black boxes and gray line, respectively. **(B)** Alignment of the deduced amino acid sequences of *BnaC.SP6* and *BnaA.SP6*. The signal peptides are indicated by thin black lines under the corresponding residues. **(C)** Detection of *Bna.SP6* transcript in *B. napus* by RT-PCR. Buds were 1.5, 2.5, 3.5, 4.5, 5.5, and 6.5 mm length; 2, 11, and 29 dpi, siliques 2, 11, and 29 days after artificial pollination.

*AT1G19500* belongs to small secreted protein gene family and named *ATSP6* (Nakamura et al., 2012). Two ATSP6 homologs, BnSP6_C08 and BnSP6_A08, were cloned from *B. napus* “ZS11.” BnSP6_C08 was observed to have a significantly longer intron than that present in its paralog, BnSP6_A08 (**Figure 1A**). Their coding sequences (CDS) were also cloned from the opening anthers of “ZS11” and designated as *BnaC.SP6* and *BnaA.SP6*. Sequence alignment showed that *BnaC.SP6* and *BnaA.SP6* only had five mismatches in their CDS sequences. They encoded 81-a.a. peptides containing putative signal cleavage sites between residues 25 and 26, and had not any discernable motif (**Figure 1B**). Thus, we used the cloning primers to analyze the full-length transcripts of *Bna.SP6* (*BnaC.SP6* and *BnaA.SP6*) by RT-PCR in different organs. The result revealed that *Bna.SP6* existed high expression levels in whole inflorescence, opening anthers, and 29 dap (dap, day after artificial pollination) siliques. The expression levels were low in 5.5 and 6.5 mm buds, and 11 dap siliques; however, the expression was rarely detected in 1.5, 2.5, 3.5, and 4.5 mm buds, 2 dap siliques, buds without stamens, leaves, and stems (**Figure 1C**).

**FIGURE 2:**
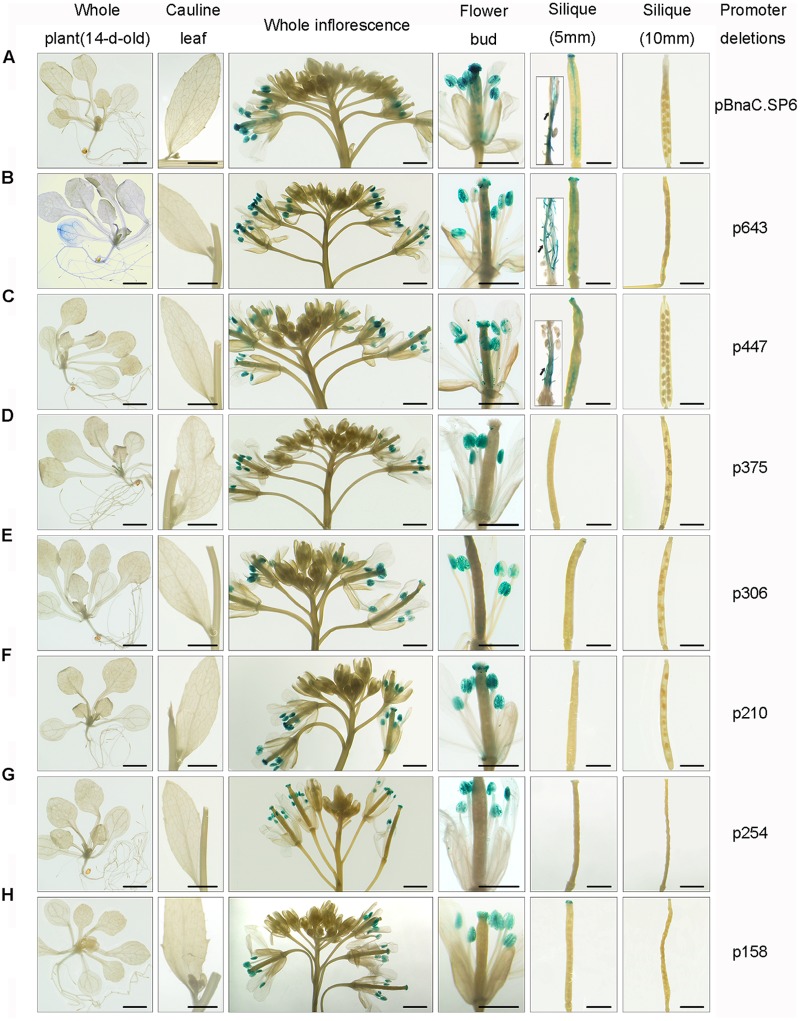
Histochemical GUS assay of pBnaC.SP6 and its deletions in transgenic *Arabidopsis*. GUS expression was analyzed in various tissues of transgenic *Arabidopsis*, namely whole plant (14-d-old), cauline leaves, whole inflorescence, flower buds, and siliques (5 and 10 mm). Black arrows indicate the silique septum. Bars, 1 mm (whole plant, cauline leaves, whole inflorescence, and siliques); 500 μm in the case of flower buds. **(A–H)** pBnaC.SP6 and its truncations (p643, p447, p375, p306, p210, p254, and p158) are shown, respectively.

In the core promoter region, pBnaC.SP6 shared 95.7% nucleic acid identity with pBnaA.SP6. So pBnaC.SP6 (1167 bp) was selected for further analysis. pBnaC.SP6 (1167 bp) was fused to GUS reporter gene, and was introduced into wild-type *Arabidopsis* (Col-0) plants. Whole plant (14-days-old), cauline leaves, whole inflorescence, flower buds, and siliques (5 and 10 mm) of transgenic lines were evaluated by GUS staining. pBnaC.SP6 showed no GUS expression in the vegetative organs, such as root and rosette leaf of 14-day-old plant. During the reproductive phase, GUS expression was observed in the anthers at flower developmental stages 11–15. Also, GUS expression was observed in the silique septum of 5 mm siliques but not in the seeds and valves (**Figure 2A**). This could be the reason as to why Bna.SP6 transcript was rarely detected in 2 dap siliques using RT-PCR (**Figure 1C**). These results indicated that *BnaC.SP6* encoded a small peptide and expressed in mature anthers and silique septum.

### Two Short Promoter Regions Independently Control the Silique Septum and Pollen Expression of *BnaC.SP6*

Motif search using PlantCARE revealed that the pollen-specific activation-related element POLLEN1LELAT52, LAT enhancer element, and GTGA MOTIF are located in pBnaC.SP6, which might help drive the expression of *BnaC.SP6*, predominantly in pollen of mature anthers (Supplementary Figure S1). Some known *cis*-elements, such as P1BS, C-box, ABRE motif, Skn_1-motif, and DOFCOREZM, were also detected. In addition, a potential TATA-box sequence was located at the position -136 (Supplementary Figure S1). Some of these elements, such as DOFCOREZM and C-box, were located around the basic promoter element, the TATA-box, and are likely to be required for the high levels of expression of *BnaC.SP6* in the developing reproductive organs.

To identify functional regions in pBnaC.SP6 (p1167), a series of promoter deletions (p643, p447, p375, p306, p254, p210, p158, and p135) cassettes were introduced into Col-0 (Supplementary Figure S2A). With 5′- and 3′-deletion constructs, GUS expression could still be detected in mature anthers (**Figures 2B–H**); however, p135 failed to show GUS activity in any of the organs tested (data not shown). p158, containing the region from -210 to -52, showed pollen-specific GUS activity, indicating that the -210 to -135 region was crucial for the expression of *BnaC.SP6* in pollen whereas the -52 to -1 region was not necessary (**Figure 2H**). Interestingly, only p643 showed weak GUS expression in the cotyledon (**Figure 2B**). When the -447 to -375 region was missing from p447, no GUS expression was observed in the silique septum (**Figures 2C,D**). This suggested that the -447 to -375 region was important for the silique septum activity of pBnaC.SP6.

### Characterization of the Pollen-Specific Activity of p158

**FIGURE 3:**
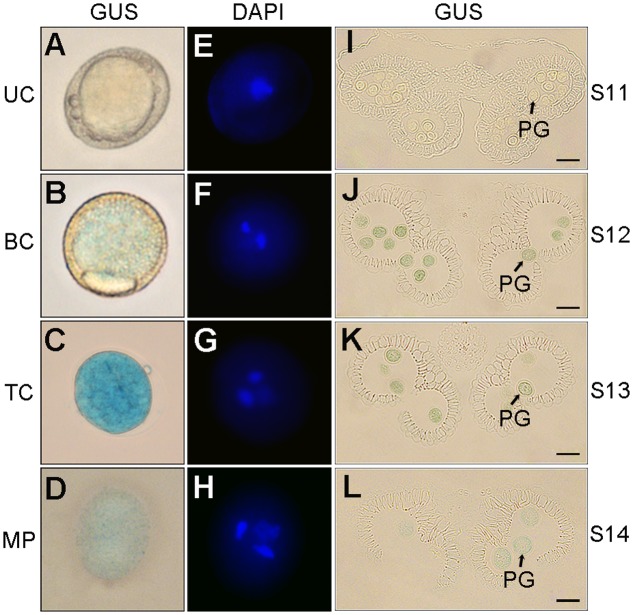
GUS expression driven by p158 in developing pollen. **(A–H)** DAPI staining of developing pollen in p158 transgenic *Arabidopsis* plants. UC, unicellular microspores; BC, bicellular pollen; TC, immature tricellular pollen; MP, mature pollen. **(I–L)** Transverse section of developing anthers of p158 transgenic *Arabidopsis* plant. PG, pollen grain. Scale bars = 20 μm.

The pollen-specific activity of p158 in developing pollen was determined using semi-thin anther sections and DAPI staining. Obvious GUS staining was first detected in the early bicellular pollen at the anther development stage 12 (**Figures 3A,B,I,J**). At stage 13, the GUS expression reached its maximal level in the tricellular pollen, and declined gradually in the pollen grains until mature pollen grains were released (**Figures 3C,D,K,L**). Similar results were obtained by staining the anthers with DAPI. The blue-fluorescing DAPI-stained nucleic acids were clearly detectable under ultraviolet illumination in the early bicellular, tricellular, and mature pollen (**Figures 3E–H**). Therefore, p158 could specifically drive the GUS expression from early bicellular pollen to mature pollen stage.

To determine the GUS activity driven by different promoter deletions of *BnaC.SP6*, quantitative GUS assays were performed after segregation analysis (Supplementary Table S2). As expect, p158 showed the minimum GUS activity in comparison to those obtained with other deleted *BnaC.SP6* promoters (Supplementary Figure S2B). The p158 region was also amplified and sequenced from *B. rapa*, *B. oleracea*, *B. juncea*, and *B. carinata*. They shared 100% nucleic acid identity with the *B. napus* p158, suggesting that the pollen-specific activity of p158 was highly conserved in these species (Supplementary Figure S3). These results indicated that the minimal promoter p158 was required for late pollen-specific expression of *BnaC.SP6* and highly conserved in *Brassica* species.

### Expression Pattern of *BnaA.bZIP1* and Its Function as a TF

**FIGURE 4:**
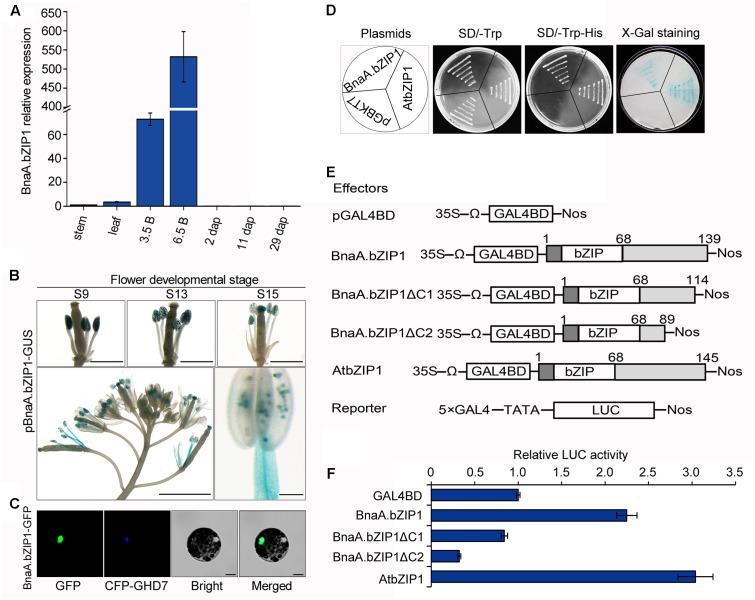
Expression pattern, subcellular localization, and the transcriptional activation activity of BnaA.bZIP1. **(A)** Expression levels of BnaA.bZIP1 in diverse tissues as assessed by qRT-PCR. Error bars indicate the SD and were calculated from three biological replicates. 3.5B, 0–3.5 mm buds; 6.5B, 3.5–6.5 mm buds; the other tissues are as mentioned in **Figure 1C**. **(B)** GUS expression in developing flower of pBnaA.bZIP1 transgenic lines. Bars, 500 μm (flower buds), 1 mm (whole inflorescence), and 100 μm in the case of stamen. **(C)** Subcellular localization of BnaA.bZIP1. The fusion plasmid (pM999-BnaA.bZIP1-GFP) and a nuclear marker plasmid (pM999-CFP-GHD7) were co-transformed into *Arabidopsis* protoplasts. Scale bars = 10 μm. **(D)** Assay for the transcriptional activation of BnaA.bZIP1 in yeast cells. The pGBKT7-AtbZIP1 and pGBKT7 were used as positive and negative controls, respectively. **(E)** Schematic diagrams of the constructs used for the transient expression assay in *Arabidopsis* protoplasts. The full-length and two C-terminal deletions of BnaA.bZIP1 were fused with GAL4BD. The pGAL4BD and pBD-AtbZIP1 were used as negative and positive controls, respectively. **(F)** The transcriptional activation abilities of effectors were determined by the ratio of LUC to REN, which was obtained from the co-transformation of protoplasts with the effector and reporter plasmids (*n* = 3). The value of the negative control was set to 1. Error bars represent the SE of three biological replicates.

Quantitative real-time PCR analysis showed that *BnaA.bZIP1* was predominantly expressed in flower buds and at low levels in stems, leaves, and 2, 11, and 29 dap siliques (**Figure 4A**). To obtain further insights into the expression pattern of *BnaA.bZIP1* in developing flowers, the pBnaA.bZIP1 reporter construct was transformed into Col-0 plants and histochemical GUS assay was performed. During flower development, the initial GUS activity was specifically detected in the anthers at flower stages from 9 to 12. From stages 13 to 15, GUS expression was also observed in the stamen filaments and perianths (**Figure 4B**). These suggested that *BnaA.bZIP1* was dynamically expressed in floral organs and coexpressed with *BnaC.SP6* in anthers. Therefore, the full-length CDS of *BnaA.bZIP1* were cloned from opening anthers. It contained a CDS of 420 bp, which encoded a 139 a.a. protein with a basic leucine zipper (bZIP) DNA-binding and dimerization domains localized between residues 17 and 68. The overall amino acid sequence identity between BnaA.bZIP1 and AtbZIP1 was 78.77% (Supplementary Figure S4). To determine the subcellular localization of BnaA.bZIP1, a vector pM999-BnaA.bZIP1-GFP was constructed by fusing GFP to the C-terminus of BnaA.bZIP1. We observed that the GFP signal coincided with the CFP signal of a previously characterized nuclear marker CCT domain protein (OsGHD7), indicating that BnaA.bZIP1 is a nuclear-localized protein (**Figure 4C**).

It had been suggested that AtbZIP1 functions as a transcriptional activator in yeast and its C-terminus is important for the transactivation activity (Sun et al., 2012). To investigate whether BnaA.bZIP1 has the similar activity as that of AtbZIP1, pGBKT7-BnaA.bZIP1, pGBKT7-atbZIP1, and pGBKT7, plasmids were separately transformed into the yeast strain AH109. All the transformants could grow well on the SD/-Trp medium, but on SD/-Trp-His medium only the yeast cells containing pGBKT7-BnaA.bZIP1 and pGBKT7-AtbZIP1 grew well. Consequently, the pGBKT7-BnaA.bZIP1 transformants showed blue color as did the colonies transformed with pGBKT7-atbZIP1 in the X-Gal staining for β-galactosidase activity (**Figure 4D**). Furthermore, the DLR assay system was used to confirm the transcriptional activation abilities of BnaA.bZIP1. The full-length BnaA.bZIP1 could activate the reporter gene but BnaA.bZIP1ΔC1 and BnaA.bZIP1ΔC2 failed to activate it, compared to the negative control pGAL4BD (**Figure 4E**). Furthermore, the positive control AtbZIP1 could also activate the reporter gene, which had almost 1.4-fold LUC activity compared to the full-length BnaA.bZIP1. These results indicated that BnaA.bZIP1 exhibited similar transcriptional activity and the C terminus of BnaA.bZIP1 was necessary for its transcription activation activity.

### BnaA.bZIP1 Acts as a Transcriptional Repressor of *BnaC.SP6*

**FIGURE 5:**
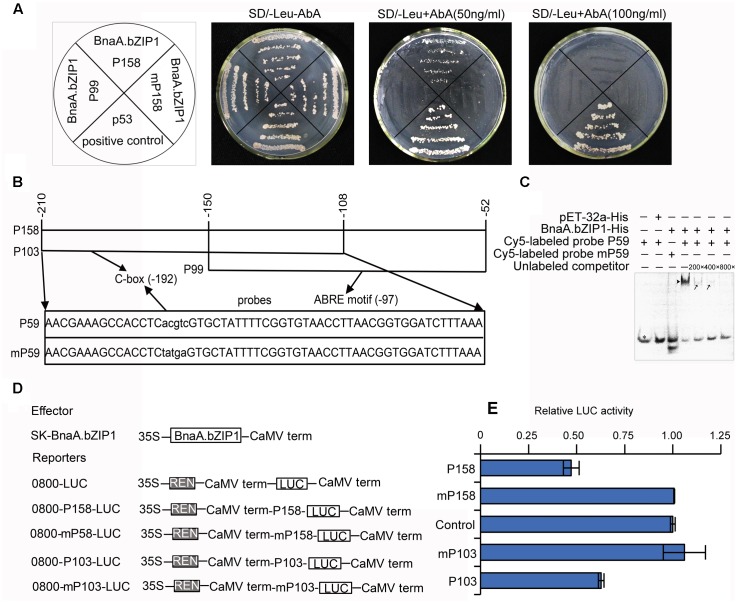
BnaA.bZIP1 binds to the p158 promoter region and acts as a transcriptional repressor. **(A)** Growth of the co-transformed yeast cells on SD/-Leu plates without or with AbA (50 or 100 ng ml^-1^). **(B)** Diagram of the p158 region. The p158 region was divided into p103 and p99 regions, with a 43-bp overlap, containing putative C-box and ABRE motifs, respectively. The p59 and mp59 probes containing the C-box or mutated C-box were used for EMSA. The core sequence (CACGTC) of the C-box was changed to Ctatga (underlined). **(C)** Binding of BnaA.bZIP1 to the C-box of p158 region in EMSA. The BnaA.bZIP1-His protein was incubated with the Cy5-labeled probe containing the C-box or mutated C-box; the pET-32a-His protein was used as a negative control; the unlabeled promoter fragment was used as a competitor in the assay. -, absence; +, presence; black triangle indicates the shifted band; black arrows indicate reduced intensity of the shifted bands; a black star indicates the free probes. **(D)** Schematic representation of the constructs used for the dual luciferase reporter (DLR) assay in *Arabidopsis* protoplasts. BnaA.bZIP1 driven by the CaMV35S promoter was used as an effector. For each reporter construct, the firefly LUC gene was driven by the p158, p103, mutated p158 (mp158), and mutated p103 (mp103) promoter, respectively. **(E)** The promoter activity was indicated by a ratio of LUC to REN as described in **Figure 4** (*n* = 3). The value of the control was set to 1. Error bars represent the SE of three biological replicates.

BnaA.bZIP1 belongs to bZIP TFs, which has been demonstrated that it can specifically recognize and bind to ACGT-containing elements in their target genes. Sequence analysis revealed that pBnaC.SP6 contained a C-box (CACGTC) and an ABRE motif (TACGTG) (Supplementary Figure S1). Moreover, *BnaA.bZIP1* was coexpressed with *BnaC.SP6* in anthers. These evidences imply that *BnaA.bZIP1* might interact to the p*BnaC.SP6*. Thus, we performed a yeast one-hybrid assay with p158, mutated p158, and p99 promoter regions to examine whether BnaA.bZIP1 could bind to the C-box or ABRE motif. All the co-transformed yeast cells grew well on SD/-Leu medium without Aureobasidin A (AbA) and the positive control p53 also grew well on SD-Leu medium with AbA (50 ng ml^-1^). Yeast cells co-transformed with pGADT7-BnaA.bZIP1 and pAbAi-p158 grew normally, but the yeast cells co-transformed with pGADT7-BnaA.bZIP1 and pAbAi-P99 or pAbAi-mp158 (with C-box sequence in the p158 changed from CACGTC to Ctatga) failed to survive on agar plates containing 50 ng ml^-1^ AbA (**Figure 5A**). This indicated that BnaA.bZIP1 specifically binds to the C-box but not to the ABRE motif in p158 promoter region in yeast.

When 100 ng ml^-1^ AbA was supplemented to the medium, only the p53 grew well on SD/-Leu medium (**Figure 5A**); thus, to further confirm the interaction of BnaA.bZIP1 with C-box, an electrophoretic mobility shift assay (EMSA) was performed with Cy5-labeled probes, p59 and mp59 (**Figure 5B**). The recombinant fusion protein, BnaA.bZIP1-His, was able to bind to the p59 probe, but failed to bind to the C-box-mutated probe, mp59. Furthermore, this specific binding could be reduced by competition with unlabeled probe at 200×, 400×, and 800× concentrations (**Figure 5C**). The negative control, pET-32a-His, failed to bind to the p59 probe. These results strongly provide further support that BnaA.bZIP1 can directly bind to the C-box in the p158 region. The results described above revealed that BnaA.bZIP1 has transcriptional activity; however, it was not clear whether it was an activator or a repressor. To determine this, a DLR assay using Arabidopsis protoplasts was performed to investigate how BnaA.bZIP1 interacts with the p158 region (**Figure 5D**). The presence of the effector 62-SK-BnaA.bZIP1 and the reporter 0800-p158-LUC resulted in a 50% reduction in LUC activity compared to that in the control, whereas such a reduction was not observed with the 0800-mp158-LUC reporter. Similar level of reduction in LUC activity was obtained for the combination of 62-SK-BnaA.bZIP1 and 0800-p103-LUC, but for 0800-mp103-LUC, the LUC activity was close to that in the control (**Figure 5E**). Thus, it was confirmed that BnaA.bZIP1 could specifically bind to the C-box sequence and suppress the p158 activity.

**FIGURE 6:**
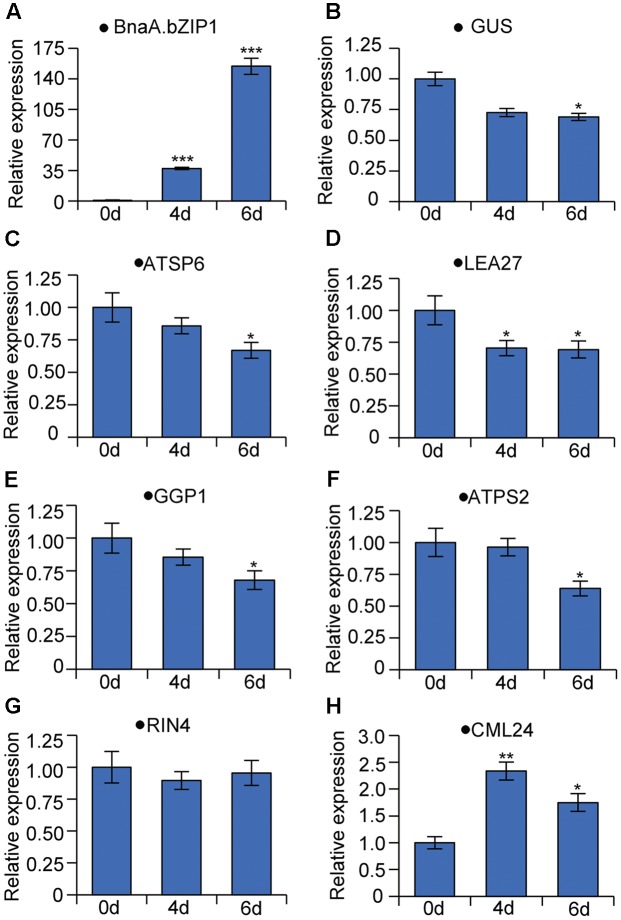
qRT-PCR analyses of genes bound and repressed by AtbZIP1 in DEX-induced BnaA.bZIP1/p158 plants. The data were calculated according to the 2^-ΔΔ*C*_t_^ method and the mRNA levels of genes on 0 day were set to 1. Bars show means ± SD (*n* = 3). The RNA samples from buds were tested on 0, 4, and 6 days after treatment with DEX. **(A–H)** Relative mRNA levels of *BnaA.bZIP1*, *GUS* (*Escherichia coli* beta-glucuronidase gene), *ATSP6* (At1g19500), *LEA27* (LATE EMBRYOGENESIS ABUNDANT 27, At2g46140), *GGP1* (GAMMA-GLUTAMYL PEPTIDASE 1, At4g30530), *ATPS2* (PHOSPHATE STARVATION-INDUCED GENE 2, At1g73010), *RIN4* (RPM1-interacting protein 4 family protein, At2g17660), and *CML24* (CALMODULIN-LIKE 24, At5g37770).

We also generated the stable transgenic plants expressing BnaA.bZIP1 with a dexamethasone (DEX)-inducible system in the background of p158 promoter transgenic line. qRT-PCR was performed to measure the suppression activity of BnaA.bZIP1 using total RNA from late buds of the DEX-inducible plants. Firstly, the expression level of *BnaA.bZIP1* was induced to 37- and 154-fold higher than that in the control (**Figure 6A**), and the expression of *GUS* was decreased by 1.5-fold as expected (**Figure 6B**), which is consistent with the transient assay, indicating that BnaA.bZIP1 suppressed the activity of p158 (**Figure 5E**). Secondly, the expression of *BnaC.SP6* homologous gene, *ATSP6*, was also decreased in the inducible plants (**Figure 6C**). Additionally, five genes (*LEA27*, *GGP1*, *ATPS2*, *CML24*, and *RIN4*) which were previously characterized to be bound and repressed by AtbZIP1, were also detected (Para et al., 2014). qRT-PCR results showed that the expression levels of these genes were consistently decreased in the inducible plants except for *CML24* and *RIN4* (**Figures 6D–H**). These results strongly supported that BnaA.bZIP1 acts as a transcriptional repressor of *BnaC.SP6* in pollen activity.

## Discussion

AtbZIP1 can directly bind to various ACGT-containing elements to regulate the expression of downstream genes (Para et al., 2014). In this study, qRT-PCR and GUS staining showed that its homologous gene, *BnaA.bZIP1* was predominantly expressed in whole anthers (**Figures 4A,B**). *BnaA.bZIP1* encoded a nuclear-localized bZIP-type DNA-binding protein and possessed transcriptional activation activity in the yeast and protoplast assay system (**Figures 4C–E**). Y1H showed that BnaA.bZIP1 could specifically bind to the C-box but not to the ABRE motif in p158 (**Figure 5A**). The ABRE motif was present in p158 but not in the -210 to -135 region, indicating that it was not necessary for the pollen-specific activity of p158. The EMSA and DLR assay results also showed that BnaA.bZIP1 interacted directly with p158 by binding to the CACGTC (C-box) element (**Figures 5C–E**). In addition, we demonstrated that BnaA.bZIP1 could suppress the p158 activity in DEX-induced BnaA.bZIP1/p158 plants (**Figure 6B**). Thus, BnaA.bZIP1 functions as a transcriptional repressor of *BnaC.SP6*, providing evidence that a bZIP TF participates in pollen development. However, further study is needed to explore whether or not C-box is crucial for the pollen-specific activity of p158. In additional, the dramatically expression of BnaA.bZIP1 in flower organs implies that other factors might contribute to pollen-specific expression of *BnaC.SP6*. The bZIPs are known to form homodimers and heterodimers for DNA-binding and regulation of transcription (Weltmeier et al., 2006; Schutze et al., 2008; Alonso et al., 2009). The dimer composition determines the outcome of target gene expression. Two bZIP proteins, EEL and ABA-insensitive 5 (ABI5) act antagonistically on the same target promoter: ABI5 homodimers activate the gene expression, whereas EEL homodimers and ABI5–EEL heterodimers suppress it (Bensmihen et al., 2002). Similarly, AtbZIP1 forms heterodimers with AtbZIP10 or AtbZIP63, which could improve its binding affinity for G-box and C-box *cis*-elements (Kang et al., 2010). Gibalova et al. (2017) also confirmed that the potential homodimerization of AtbZIP18/AtbZIP18, AtbZIP28/AtbZIP28, AtbZIP60/AtbZIP60, and heterodimerization of AtbZIP61/AtbZIP18, AtbZIP18/AtbZIP34, bZIP28/bZIP60 by Y2H assays among the pollen-expressed bZIP TFs. AtbZIP18 possessed high dimerization capacity and acted as a repressor with its EAR (ethylene-responsive element binding factor-associated amphiphilic repression) motif in pollen development network. Furthermore, the transactivation activity of the same bZIP can be further modified through its interaction with other proteins. HY5 (ELONGATED HYPOCOTYL 5) is enhanced by the clock protein CCA1, but is inhibited when it interacts with BBX25 (Andronis et al., 2008; Gangappa et al., 2013). Other TFs family could also form homodimers or heterodimers to regulate downstream gene expression, such as MADS-box TFs of the MIKC^∗^ class. For example, AGL65, AGL66, AGL94, and AGL104 could form three heterodimers and repress early genes at late stages of pollen development (Adamczyk and Fernandez, 2009). In our study, BnaA.bZIP1 possessed transcription activation activity but suppressed the pollen activity of *BnaC.SP6*. These results imply that BnaA.bZIP1 might interact with other bZIPs to suppress the expression of *BnaC.SP6* by binding to the C-box.

*Arabidopsis* has been widely used to effectively characterize the *cis*-elements of the anther/pollen-specific promoters for homologous and heterologous crop plant species (Swapna et al., 2011; Kim et al., 2014; Gao et al., 2016). In our study, the activities of pBnaC.SP6 deletions were analyzed in *Arabidopsis*. The discrete reduction in GUS activity of the pBnaC.SP6 deletion constructs implies that the *cis*-elements are complicated (Supplementary Figure S2B). We showed that the -447 to -375 and -210 to -135 regions are essential for the silique septum and pollen expression of *BnaC.SP6*, respectively (**Figure 2**). The -447 to -375 region lacks any known *cis*-acting elements required for silique septum-specific expression. One putative Skn_1-motif is absent in the -447 to -375 region. However, there was nearly fourfold reduction in GUS activity in the case of p375 where we deleted the -447 to -375 region (Supplementary Figure S2B). This suggests that some novel *cis*-acting elements, which can control *BnaC.SP6* expression in the silique septum, are located within the -447 to -375 region. Nguyen et al. (2016) identified that γMYB1 binds to the P1BS *cis*-element, and activates the expression of PLA2-γ with the assistance of its co-activator, γMYB2. When we deleted the -306 to -210 region containing a P1BS, p210 exhibited a 1.90-fold decrease in GUS activity compared to that observed for p306. This implies that P1BS contributes to the pollen activity of p306, which might also be activated by γMYB1.

In this study, p375, p254, and p210, which were confirmed to be anther-specific promoters in Arabidopsis, could be used in generating the transgenic male sterility lines in *B. napus*. In addition, Chang et al. (2016) constructed a male sterility system for hybrid breeding and seed production in rice using three modules consisting of a restorer gene, *OsNP1*, the red fluorescence protein gene, *DsRed*, and the maize α-amylase gene, *ZM-AA1*, under the pollen-specific PG47 promoter (Chang et al., 2016). S45AB of rape (*B. napus* L.) is a recessive genic male sterile line just like the osnp1-1 mutant of rice. Therefore, similar seed production technology system of S45AB can be constructed in *B. napus* using the late pollen-specific promoter p158 fusing with *ZM-AA1* to specifically kill the 50% transgenic pollen. However, further analyses are needed.

## Conclusion

We confirmed that two short promoter regions independently control the specificity of *BnaC.SP6*. BnaA.bZIP1 could interact to C-box and function as a transcriptional repressor of *BnaC.SP6*. These findings provide new insight into the regulator of *BnaC.SP6* and the pollen/anther-specific regions of pBnaC.SP6, which should have potential application in genetic manipulation. However, further evaluation is needed for use in the genetic engineering of *B. napus*.

## Author Contributions

JT and XW designed the study. XW prepared materials, performed most of experiments, and wrote the original manuscript. Other authors assisted in experiments and discussed the results.

## Conflict of Interest Statement

The authors declare that the research was conducted in the absence of any commercial or financial relationships that could be construed as a potential conflict of interest.
