# Supplementary material for: BnaA.bZIP1 Negatively Regulates a Novel Small Peptide Gene, BnaC.SP6, Involved in Pollen Activity
Source: Front Plant Sci. 2017 Dec 12;8:2117. doi: 10.3389/fpls.2017.02117 (PMC5732959; doi:10.3389/fpls.2017.02117)
Supplement: Supplementary file 1 [file Presentation_1.PDF]

Supplementary Material

Figure S1. Putative cis-acting elements in pBnaC.SP6.

Figure S2. Analysis of the activities of pBnaC.SP6 deletions in transgenic *Arabidopsis* plants.

Figure S3. Homology analysis of p158 regions from *Brassica napus, B. juncea*, *B. carinata*, *B. rapa*, and *B. oleracea*.

Figure S4. Alignment of the deduced amino acid sequences of BnaA.bZIP1 and AtbZIP1.

Table S1. Primers used in the study.

Table S2. *Arabidopsis* lines transformed with different promoters showing 3:1 segregation in T_2 generation.

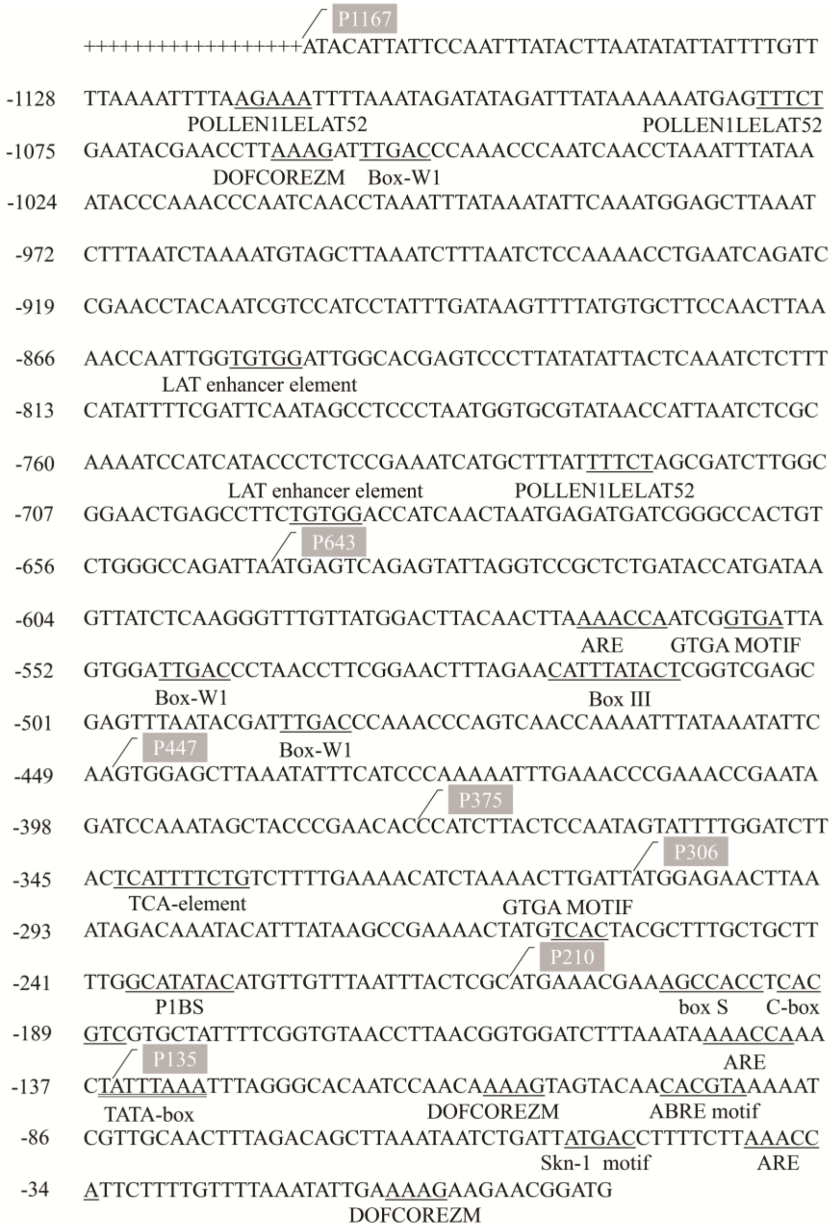

Figure S1. Putative cis-acting elements in pBnaC.SP6.

The cis-acting elements are underlined. The putative TATA-box is underlined with double lines.

The positions of the promoter deletions are indicated by linear dimensions.

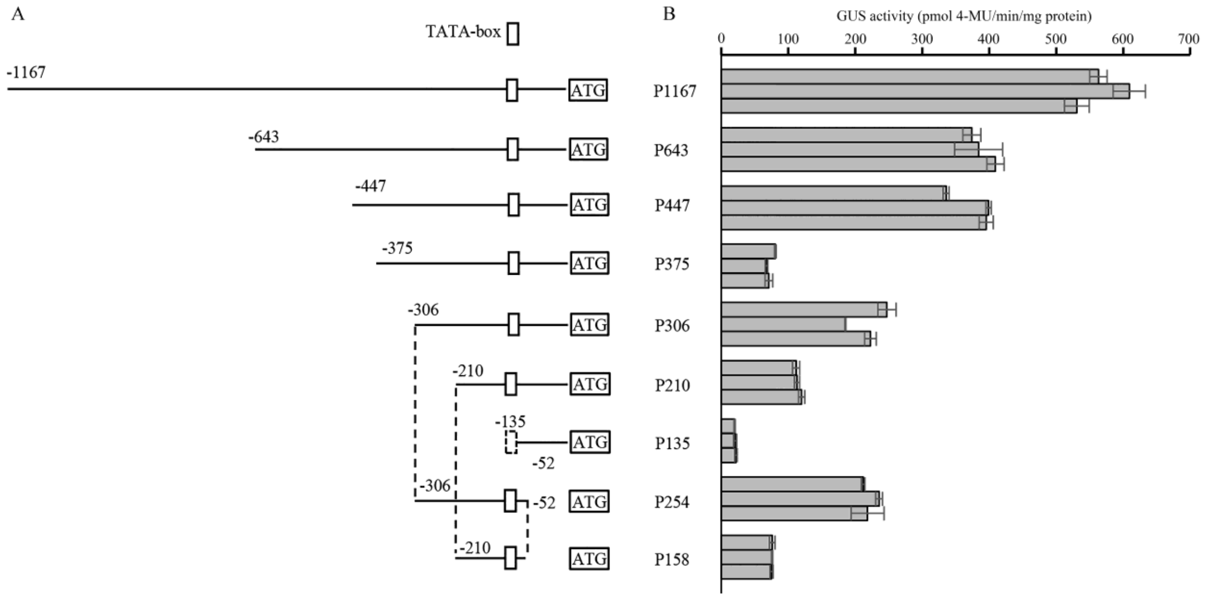

Figure S2. Analysis of the activities of deletion mutants of pBnaC.SP6 in transgenic *Arabidopsis* plants.

(A) Schematic of pBnaC.SP6 promoter deletions. The numbers of nucleotides counted upstream from the translation start codon of *BnaC.SP6* were shown.

(B) Corresponding GUS activity driven by pBnaC.SP6 and its deletions in transgenic *Arabidopsis*. GUS activity was determined using protein extracts from buds (flower development stage 12 to 15). Samples were harvested from 3 transgenic lines with three individual plants of each line, respectively. Error bars represent the SE of three biological replicates.

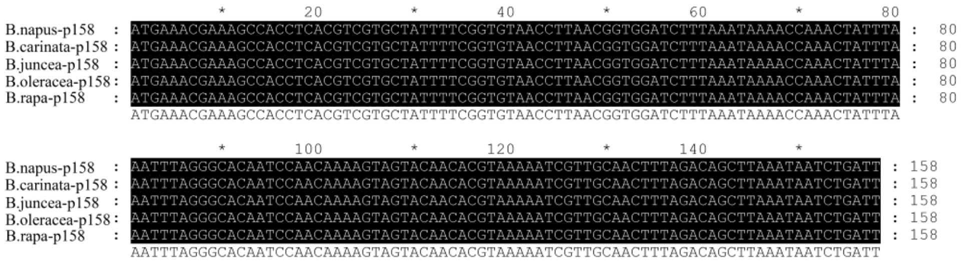

Figure S3. Homology analysis of p158 regions from *Brassica napus*, *B. juncea*, *B. carinata*, *B. rapa*, and *B. oleracea*. All p158 sequences were obtained from clones sequencing.

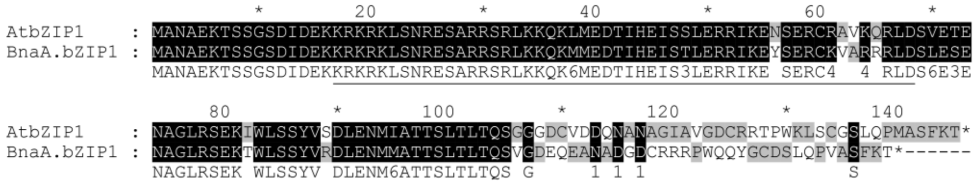

Figure S4. Alignment of the deduced amino acid sequences of BnaA.bZIP1 and AtbZIP1. The bZIP DNA-binding and dimerization domain is indicated by thin black line under the corresponding residues.

Table S1. Primers used in this study

Primer name	Sequence(5’-3’)	Use
Bna.SP6-F	ATGGCGAAAACATGGGTAGCT	gDNA、CDS and RT-PCR for Bna.SP6
Bna.SP6-R	TTAGAAATGAATTCCCATTTCCTTG	
BnUBA-F	TGGACATCCCAGTTTCAACA	
BnUBA-R	CTGAAGGACGGCAAAGAAAG	
p1167F	CGCGTCGACATACATTATTCCAATTTATACTTAA	5’-deletion and 3’-deletion promoter constructs of BnaC.SP6
p643F	CGCGTCGACATGAGTCAGAGTATTAGGTCCGC	
p447F	CGCGTCGACGTGGAGCTTAAATATTTCATCCC	
p375F	CGCGTCGACCCATCTTACTCCAATAGTATTTTGG	
p306F/ p254F	CGCGTCGACATGGAGAACTTAAATAGACAAATAC	
p210F/p158F	CGCGTCGACATGAAACGAAAGCCACCTC	
p135F	CGCGTCGACATTTAAATTTAGGGCACAATCC	
Pro-1R	CCCCGGGCCGTTCTTCTTTTCAATATTTAAAA	
Pro-d52R	CCCCGGGAATCAGATTATTTAAGCTGTCTAAA	
pAbAi-p158-F	TGCAAGCTTATGAAACGAAAGCCACCTC	Yeast One-Hybrid
pAbAi-p158-R	CGCGTCGACAATCAGATTATTTAAGCTGTCTAAA	
pAbAi-mp158-F	TGCAAGCTTATGAAACGAAAGCCACCTCTATGAGTG	
pAbAi-p99-F	TGCAAGCTTTAAATAAAACCAAACTATTTAAATT	
AD-BnaA.bZIP1-F	AGCCATATGATGGCAAACGCTGAGAAGACA	
AD-BnaA.bZIP1-R	CACTCGAGTGTCTTGAAAGACGCAACTG	
p59-CY5-F	AACGAAAGCCACCTCACGTCGTGCTATTTTCGGTGT AACCTTAACGGTGGATCTTTAAA	Electrophoretic mobility shift assays between BnaA.bZIP1 and C-box of p158
p59-R	TTTAAAGATCCACCGTTAAGGTTACACCGAAAATAGC ACGACGTGAGGTGGCTTTCGTT	
mp59-CY5-F	AACGAAAGCCACCTCTATGAGTGCTATTTTCGGTGTA ACCTTAACGGTGGATCTTTAAA	
mp59-R	TTTAAAGATCCACCGTTAAGGTTACACCGAAAATAGC ACTCATAGAGGTGGCTTTCGTT	
32a-BnaA.bZIP1-F	GCGGATCCATGGCAAACGCTGAGAAG	His-fused protein expression
32a-BnaA.bZIP1-R	CACTCGAGTGTCTTGAAAGACGCAACTG	

BD-AtbZIP1-F	AGCCATATGATGGCAAACGCAGAGAAGACA	Transcription activation assay in yeast cells
BD-AtbZIP1-R	GCGGATCCTGTCTTAAAGGACGCCATTGGT	
BD-BnaA.bZIP1-F	AGCCATATGATGGCAAACGCTGAGAAGACA	
BD-BnaA.bZIP1-R	GCGGATCCTGTCTTGAAAGACGCAACTG	
GAL4-AtbZIP1-F	GCTCTAGAATGGCAAACGCAGAGAAGACAA	Analyses of BnaA.bZIP1 transcriptional activation/repression and DNA binding in Arabidopsis protoplasts
GAL4-AtbZIP1-R	GCGGATCCTCATGTCTTAAAGGACGCCATTG	
GAL4-BnaA.bZIP1-F	GCTCTAGAATGGCAAACGCTGAGAAGACA	
GAL4-BnaA.bZIP1-R	GCGGATCCTCATGTCTTGAAAGACGCAACTG	
GAL4-BnaA.bZIP1-N-2R	GCGGATCCTCATCTAACGTAGCTTGAAAGCC	
GAL4-BnaA.bZIP1-N-3R	GCGGATCCTCACTCGCTGTACTCTTTGATT	
SK-BnaA.bZIP1-F	GCGGATCCATGGCAAACGCTGAGAAG	
SK-BnaA.bZIP1-R	CACTCGAGTGTCTTGAAAGACGCAACTG	
0800-p158-F (0800-p103-F)	CGCGTCGACATGAAACGAAAGCCACCTC	
0800-p158-R	CCCCGGGAATCAGATTATTTAAGCTGTCTAAA	
0800-mp158-F (0800-mp103-F)	CGCGTCGACATGAAACGAAAGCCACCTCTATGAGTG	
0800-p103-R	CCCCGGGTTTGTTGGATTGTGCCCTAA	
BnaA.bZIP1-qp-158F	TCAAAGAGTACAGCGAGAGATGC	qRT-PCR for BnaA.bZIP1 in *B. napus*
BnaA.bZIP1-qp-304R	GCGTTAAGGAAGTCGTAGCCA	
999-BnaA.bZIP1-F	GCTCTAGAGCCACCATGGCAAACGCTGAGAAG	Subcellular localization of BnaA.bZIP1
999-BnaA.bZIP1-R	GCTCTAGATGTCTTGAAAGACGCAACTG	
pBnaA.bZIP1-F	CGCGTCGACATCCGAAAAAGTAGGCTGGG	Promoter of BnaA.bZIP1
pBnaA.bZIP1-R	CGGGATCCATTTTGTCCTAACACTTTGCGAG	
702-BnaA.bZIP1-F	CGCGTCGACATGGACTATAAGGACCACGACG	pTA-3XFlag-BnaA.bZIP1 construct
702-BnaA.bZIP1-R	CGACTAGTTCATGTCTTGAAAGACGCAACTG	

Flag-BnaA.bZIP1-qp-F	CCACGACGGAGACTACAAGGAT	
Flag-BnaA.bZIP1-qp-R	TTGCCTCCTGTTCATCGC	
GUS-qp-F	CTGCGGTTAGACTTGTGTTGC	
GUS-qp-R	TTCCAGTCCTTTCCCGTAGTC	
ATSP6-qp-F	GTTGTCGGTAATGCTACTTGTCTC	
ATSP6-qp-R	TAGAAGTGGATTCCCCATTCC	
LEA27-qp-F	GGAGTAGATTATCACGCCAAGGT	
LEA27-qp-R	TTCATCAGACTAACCGCTATGCTAT	
GGP1-qp-F	TTCTTGGCATCTGCTTTGGTC	
GGP1-qp-R	ACTTCGTCCTGGTGACATTTGAT	Primers for qPCR in the DEX-induced plants
ATPS2-qp-F	TCAATCAACTTCTCCCCACCA	
ATPS2-qp-R	TGTTTGCATCGCTCACTATCCTA	
CML24-qp-F	TCGGAGGAGGAGGTAACAATC	
CML24-qp-R	ACAGAGCACTTCTCACCCAAA	
RIN4-qp-F	TTCGGGGAATGGGATGTGA	
RIN4-qp-R	TAAGCAAAAGTGAAACAGAGCCAT	
At-Actin7-F	GGAACTGGAATGGTGAAGGCTG	
At-Actin7-R	CGATTGGATACTTCAGAGTGAGGA	

***Underlined sequences represent restriction enzyme recognition sites. F, forward primer; R, reverse primer.**

Table S2. Promoter transformants of *Arabidopsis* showing 3:1 segregation in T_2 generation.

Promoter region	Progenies of transformants	Inoculated	Germinated	Resistant to kanamycin	Sensitive to kanamycin	$(\lvert O-E \rvert-0.5)^2/E$	P vaule
p1167	3	147	147	112	35	0.057	0.812
	6	149	149	111	38	0.002	0.962
	7	127	127	94	33	0.024	0.878
p647	1	159	158	118	40	0.000	1.000
	6	126	126	94	32	0.000	1.000
	30	135	135	100	35	0.022	0.881
p447	2	132	132	99	33	0.010	0.920
	4	149	149	114	35	0.110	0.741
	5	120	120	90	30	0.011	0.916
p375	2	148	148	112	36	0.009	0.924
	10	130	130	97	33	0.000	1.000
	28	146	146	112	34	0.146	0.702
p306	11	135	135	101	34	0.002	0.960
	28	159	159	118	41	0.019	0.891
	34	132	132	99	33	0.010	0.920
p210	4	114	114	86	28	0.000	1.000
	8	129	127	97	30	0.066	0.798
	9	134	134	100	34	0.000	1.000
p135	1	131	131	99	32	0.003	0.960
	8	152	152	113	39	0.009	0.925
	11	160	159	119	40	0.002	0.963
p254	2	130	130	97	33	0.000	1.000
	5	136	136	101	35	0.010	0.921
	8	150	150	111	39	0.036	0.850
p158	10	103	103	78	25	0.003	0.955
	13	118	118	90	28	0.045	0.832
	14	112	112	84	28	0.012	0.913
